# Challenges in natural product-based drug discovery assisted with *in silico*-based methods

**DOI:** 10.1039/d3ra06831e

**Published:** 2023-10-30

**Authors:** Conrad V. Simoben, Smith B. Babiaka, Aurélien F. A. Moumbock, Cyril T. Namba-Nzanguim, Donatus Bekindaka Eni, José L. Medina-Franco, Stefan Günther, Fidele Ntie-Kang, Wolfgang Sippl

**Affiliations:** a Center for Drug Discovery, Faculty of Science, University of Buea P.O. Box 63 Buea CM-00237 Cameroon veranso.conrad@gmail.com; b Structural Genomics Consortium, University of Toronto Toronto Ontario M5G 1L7 Canada; c Department of Pharmacology & Toxicology, University of Toronto Toronto Ontario M5S 1A8 Canada; d Department of Chemistry, University of Buea Buea Cameroon; e Department of Microbial Bioactive Compounds, Interfaculty Institute for Microbiology and Infection Medicine, University of Tübingen 72076 Tübingen Germany; f Institute of Pharmaceutical Sciences, Albert-Ludwigs-Universität Freiburg Freiburg Germany; g DIFACQUIM Research Group, Department of Pharmacy, School of Chemistry, Universidad Nacional Autónoma de México, Avenida Universidad 3000 Mexico City 04510 Mexico; h Institute of Pharmacy, Martin-Luther University Halle-Wittenberg Halle (Saale) Germany wolfgang.sippl@pharmazie.uni-halle.de

## Abstract

The application of traditional medicine by humans for the treatment of ailments as well as improving the quality of life far outdates recorded history. To date, a significant percentage of humans, especially those living in developing/underprivileged communities still rely on traditional medicine for primary healthcare needs. *In silico*-based methods have been shown to play a pivotal role in modern pharmaceutical drug discovery processes. The application of these methods in identifying natural product (NP)-based hits has been successful. This is very much observed in many research set-ups that use rationally *in silico*-based methods in combination with experimental validation techniques. The combination has rendered the use of *in silico*-based approaches even more popular and successful in the investigation of NPs. However, identifying and proposing novel NP-based hits for experimental validation comes with several challenges such as the availability of compounds by suppliers, the huge task of separating pure compounds from complex mixtures, the quantity of samples available from the natural source to be tested, not to mention the potential ecological impact if the natural source is exhausted. Because most peer-reviewed publications are biased towards “positive results”, these challenges are generally not discussed in publications. In this review, we highlight and discuss these challenges. The idea is to give interested scientists in this field of research an idea of what they can come across or should be expecting as well as prompting them on how to avoid or fix these issues.

## Abstract 2

This review uses documented studies to highlight current difficulties faced by natural product drug discovery scientists using *in silico* methods. This will provide interested scientists in this field of research an idea of what they can expect.

## Graphical abstract

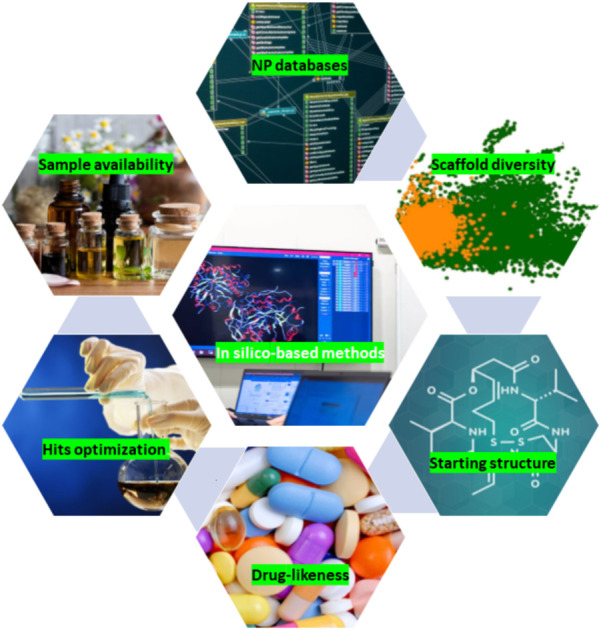

## Introduction

Communicable and non-communicable diseases continue to be a burden, causing serious affliction to diverse populations globally.^1,2^ Throughout history, humans have treated diseases and improved the quality of life by applying and using traditional medicine. Knowledge of the preparation or consumption (in several forms) was initially self-taught or passed through generations *via* word of mouth. It is important to note that formal training in quality control of these natural products (NPs) was not taken into account.^3^ One possible means of documenting and safeguarding the information and/or standardizing and improving the quality of traditional products being consumed is through scientific exploration/validation of the known traditional methods and the source species.

**Fig. 1:**
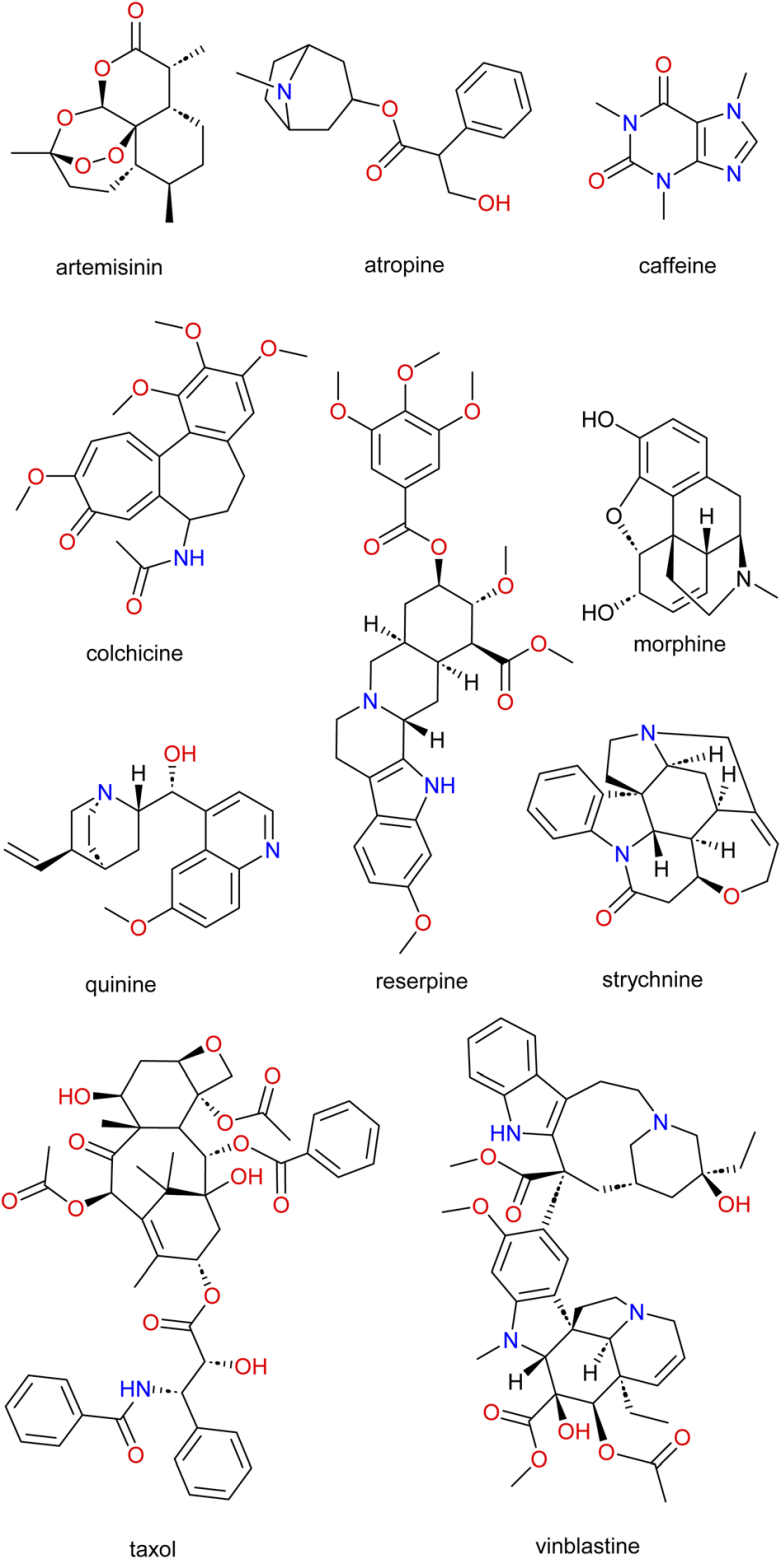
Examples of well-known NPs.

The richness of the world's flora and fauna is still being explored by *c.a.* 80% of the population in developing countries as a primary source of healthcare and needs.^4^ This high percentile dependence of the population can be attributed to socioeconomic reasons, cultural practices, personal beliefs or the difficulty in accessing modern pharmaceutical products, many times associated with the high costs of the latter. This further confirms a shift from synthetically produced medications to the use of NPs.^5–8^ Although the first pure NPs (*e.g.*, atropine, colchicine, morphine, and strychnine; Fig. 1) were isolated in the early 1800s, it was not until the 1950s that modern medicine and pharmaceutical industry turned toward plants, combinatorial chemistry, and high throughput screening; especially for anticancer drug discovery.^9–11^ Nowadays, it is evident that global and international marketing of traditional medicinal products has received attention partly due to the popularity, usage, economic value and importance of traditional medicines.^12,13^

### Role of NPs in traditional medicine

The use of traditional medicine in treating diseases throughout history is undebatable and has equally continued to provide a significant contribution to modern medicine. New strategies to search, identify, and develop new drug molecules void of resistance and side effects as well as being cheaper are needed. The revisiting of NPs is one such strategy.^14^ NPs are isolated from diverse organisms (bacterial, fungi, plants, or animal species). They equally have proven to be a good starting point for the search for pharmacologically active compounds (*e.g.*, caffeine (*Coffea* spp.), morphine (*Papaver somniferum*), nicotine (*Nicotiana tabacum*), antimalarial drugs such as quinine (*Cinchona* spp.) and artemisinin (*Artemisia annua*), reserpine (*Rauvolfia serpentina*) and the anticancer drugs Taxol (*Taxus brevifolia*) and vinblastine (*Catharanthus roseus*); Fig. 1) against several ailments.

A broad range of fields such as medicinal chemistry and drug discovery, ecology, biosynthesis and chemical biology, among others, are demonstrating interest in a deeper understanding of NP resources. Likewise, several exciting and new technologies for NP drug discovery for example “smart screening” methods, robotic separation with structural analysis, metabolic engineering, and synthetic biology have seen an absolute increase in recent years.^15–18^ These exciting and improved strategies coupled with advances in technologies can, therefore, act as a reliable means to acquire more NPs as well as their samples for biological screening.^14,19–21^ The acquiring of more NPs is in line with what several studies have reported about the advantages of NPs over combinatorially synthesized compounds in the search of biologically relevant and privileged scaffolds.^22–33^ Principal component analysis (PCA) between NPs, marketed drugs, and synthetic compounds confirmed the aforementioned statement by revealing that combinatorial compounds covered a well-defined and restricted area while drugs and NPs occupied approximately the same chemical space—covering almost all of the combinatorial compounds' diversity space as well as a much larger additional volume.^17,20,21,30–39^ Designing such NP analogues being inspired by nature is almost impossible, thus, it is necessary to emphasize that the place of NPs remains unique. This uniqueness can therefore be a very helpful step in answering questions such as: (i) which compounds should be prepared? (ii) How should they be prepared (*e.g.*, biological or diversity-oriented synthesis)? and (iii) How should such a biological/diversity-oriented synthesis be planned?^17,22–24,40–42^

### NP databases and *in silico*-based methods

Low hit rates after screening large synthetic combinatorial databases left drug discoverers the choice to either increase the diversity of combinatorial databases through improved diversity of synthetic reaction(s) and/or make a return to NPs which have worked in the past.^43^ Historically, NPs have played a significant role in drug discovery, particularly for cancer and infectious diseases. Due to their high structural complexity and diversity, they provide new lead compounds possessing unique scaffolds.^14,17,18,20,21,29,34,35,37,44–46^ The renewed interest in NPs has invited huge investment in the search (isolation, characterization, and biological evaluation) of NPs from both academia and industrial sectors.^14,19^ These efforts resulted in an increased number of NPs being isolated, characterised, and reported in the literature.^14^ NP databases and repositories stand out as one of the major means of safeguarding the collections as well as documenting and sharing the findings.^47^ There are many examples of these NP databases; from comprehensive^47–49^ (including compounds from terrestrial, marine and microbial organisms) to focussed ones^50,51^ (based on a particular disease, or compounds from specific geographical regions or organism types).^47,52–54^

The application of *in silico*-based methods^55^ to explore NP databases to identify potent hit molecules that can be optimized has seen many successful cases.^46,56–59^ They have played a pivotal role in both pharmaceutical industries and academia during the last three decades and have dramatically reduced the cost factors associated with introducing new drugs to the market.^59–63^ As an example, virtual screening (VS) procedures (which are useful in narrowing down the number of molecules for experimental testing in biological assays) with/out the combination of other more rigorous *in silico*-based methods (such as molecular dynamics and binding free energy predictions) are widely used in identifying NP based hit molecules.^58–60,62,64^ Likewise, accumulated data for NPs have also prompted the growing use of machine learning techniques in several NP drug discovery studies, from the detection of a biosynthetic gene cluster (BGC) to functional annotation of NPs.^65,66^ Moreover, poor pharmacokinetic profiles, usually discovered at late stages of the drug discovery pipeline (which could be avoided with the help of *in silico*-based predictions) has prevented many molecules from entering the market.^67^ Hence, the early computational prediction of absorption, distribution, metabolism, elimination, and toxicity (ADMET) profiles of NP molecules in the drug discovery pipeline has gained increased usage; as well as reducing the cost and the time factor involved for ADMET profiling using standard experimental approaches.^67–71^ Thus, *in silico*-based approaches are important for rational drug design-based methods to develop NP analogues with acceptable ADMET properties.^17,22–24^

### Contribution of NPs to modern drug discovery

The global ravages of diseases alongside the challenges of finding drugs with minimal side effects as well as the search for cheaper drug candidates that can treat these diseases, is still a huge challenge to the scientific community. Thus, innovative strategies such as revisiting nature (NPs) which had worked in the past (Fig. 1) can revolutionize and lead to the discovery of novel and potent drugs. Moreover, even with the backdrop of the diminished focus on NPs by the major pharmaceutical companies, scientific studies has shown that NPs still account for about half of the drugs approved by the US Food and Drug Administration (FDA), especially antibiotic and anticancer molecules.^44,45,72–79^

### Review focus

Historically, traditional medicine has been used to treat diseases. The application of these traditional medicinal methods still accounts for the primary source of health care for millions of people. However, the decline in interest by pharmaceutical industries/companies due to challenges such as technical barriers to screening, isolation, characterization and optimization encountered in NPs drug discovery were observed in the 1990s and onwards. Nowadays, several published papers have highlighted the successes of using *in silico*-based methods in NP drug discovery.^43,80–82^ Interestingly, the advent of *in silico*-based approaches which have contributed to boosting the identification of NP hit molecules also comes with some inflated expectations and disappointments.^62,82^ So far, there is a lack of comprehensive reviews on these challenges, for the scientific community (especially, for early career researchers in this area of research) to access.^83^

In this review, we highlight the most relevant challenges in the area of *in silico*-based drug discovery approaches when working on NPs. This will be grounded in a selection of reported challenges encountered during the quest for vital therapeutic agents. We will delve into these challenges through case studies, focusing on specific instances that have been highlighted. The limitations of *in silico*-based methods have not been included in this review and we would refer the readers to papers that cover that aspect in detail.^84,85^

## Source species and NP databases

Both academia and industry are continually showing a growing interest in NPs as a source for the development of novel and potent molecules or scaffolds for investigation as drug candidates. Investments in this area of NPs have led to the publication of many new molecules, with a good number of the newly published molecules attributed to some positive effects for treating several diseases.^48^ This rekindled interest in NPs has led to exponential growth in the number of NPs being isolated and characterized. Thus, directly related to the uncontrollable growth in NP databases as a means of sharing information with the scientific community.^47^ However, this comes with certain challenges for *in silico*-based drug discovery scientists working on NPs. In the following subsections, the challenges in the development, curation, access, and maintenance of NP databases as well as the problem of choosing from the multitude of NP databases available shall be discussed.

### Development of NP databases

This stage is met with challenges that need the help of specialists that are becoming rare and their activities cannot be automated.^86,87^ Examples of such specialized input include the processes of source species collection and identification, as well as documentation of herbarium information. The problem with source species/organisms is becoming even more challenging with the rate at which natural habitats are rapidly being destroyed amongst other factors.^7,88^ As of now, an all-inclusive and exhaustive freely accessible database for NPs does not exist. Most of the available NP databases are focus-based, for example on either some particular source organisms,^51^ geographical locations,^48,49^ targeted diseases and/or traditional uses.^49,50,89^ Additionally, no standard protocols have been established for processing and curating available information. Nevertheless, for this to be established, it is going to be another contest on its own, in the quest for whom/which group is going to lead in that aspect. Although this might look difficult, it is feasible and can be achieved; through consolidation and sharing of information *via* an open platform as proposed by Rutz *et al.*^52^ This will enhance and lead to a strong transformative potential for NPs research and beyond. The next challenge in the construction of NP databases after collecting and processing information is the issue of accessibility and maintenance of the databases. Accessibility through web servers that are active and continued over a long period is important. Mitishamba^90^ is an example of a reported NP database where accessing information is impossible because the provided web links are at present broken or dead. Further accessibility issues are linked to databases that are built for commercial purposes or are not open access.^46,47,82^ This brings us to the issue of timely maintenance/updating of the provided information. This most often is linked to public databases where funding issues are regularly encountered. Plus, questioning what is considered before an update, is it based on the quantity/quality of new data? A defined time interval? Or what else?

### Case study I: the exponential growth of information to be included in NP databases

As aforementioned, NP databases are growing and/or being published with no unified style in their design, construction and/or development like there is for protein sequence and information (UniProt; https://www.uniprot.org),^91,92^ protein structure (Protein Data Bank, PDB; https://www.rcsb.org)^93^ or curated classification and nomenclature for organisms (NCBI Taxonomy; https://www.ncbi.nlm.gov/taxonomy).^94^ With the continuous investment in the area of phytochemicals, a huge amount of data has been generated. However, translating this data into NP databases has seen exponential growth in the content/data information for NP databases, leading to the development of many database systems. The numerous NP databases, however, do not address certain very basic challenges including dereplication^46^ or cover a significant part of NP resources.^12^ An exemplified case is that of the NP ATLAS (https://www.npatlas.org); a resourced database maintained at the Simon Fraser University in Canada, focusing on microbial NPs.^95,96^ Although the database is actively updated, moving from the first version (published in 2019)^95^ to the recent one (published in 2021)^96^ saw a great deal of improvement and inclusion in terms of content/information (such as the application programming interface (API), taxonomic descriptions, and chemical ontology amongst others) curated for its end users.

### Case study II: choosing NP databases

The area of NP research continues to receive a lot of attention as depicted by the publication of new scientific articles every week to demonstrate the positive effects of NPs on the healing process of various human and animal diseases. This has led to uncontrollable growth in the number of published NP databases.^46,47,97^ In 2020, Sorokina and Steinbeck^47^ published a comprehensive review of published NP databases, indicating that less than 50% (of the overwhelming >120) of such resources published and re-used since the year 2000 were open access. It is therefore a real challenge to find a complete, comprehensive, and open-access NP database since available NP databases are mostly constructed to target particular regions,^48,98,99^ diseases,^50^ species,^51^*etc.* Therefore, several challenges including data redundancy between the different available databases, poor metadata quality in these databases as well as missing links to other vital databases like the target- and pathogen-centred databases need to be addressed. Addressing them will reduce bias in the exploration of information and increase the connection between chemistry- and biology-centred resources.

## Scaffold diversity

*In silico*-based drug design methods have gained significant applications in the exploration of the uncharted chemical space of small molecules, when searching for new hits in the drug discovery process. However, several questions like which sections of the chemical space should be investigated to identify potential drug candidates? Should chemical space of interest be focused/narrowed to synthetic, NP or pseudo-natural products to identify potential hits? Interestingly, the unique features of NPs (such as enormous scaffold diversity and structural complexity characterised by higher molecular mass, a larger number of sp^3^ carbon atoms and oxygen atoms, but fewer nitrogen and halogen atoms, higher numbers of H-bond acceptors and donors, lower calculated octanol–water partition coefficients, and greater molecular rigidity compared with synthetic compound libraries)^100,101^ when compared to synthetic compounds can be explored for innovative solutions in the search of novel therapeutic agents for the treatment of diseases.^44,46,47,51,52,59,77,80,102^ Thus, the unique features of NPs have offered pharma industries and academic research groups opportunities to focus on cutting-edge computational technologies to facilitate the identification of novel NP-based hits. However, several challenges including expansion of the searchable drug-like chemical space, and revisiting neglected, or non-traditional chemical spaces are encountered in this process. Also, limitations in the synthetic routes required to obtain the complex structures of NPs as well as the laborious process involved in the isolation of a single chemical constituent, usually in low yields are also various types of difficulties encountered in wet labs.^103^ An example of a study that illustrates the hurdles that could be encountered with the diversity of NPs in *in silico*-based drug discovery is presented below.

### Case study I: an example of a challenge observed with NPs diversity

The spatial arrangement, different configurations, three-dimensional molecular shape and ring complexity of NPs constitute a few of the observed structural complexity. In addition to the structural complexity, the limited amount of biological data makes it challenging to develop *in silico*-based methods that are focused on NPs. The development and application of such *in silico*-based methods and algorithms for NPs will necessitate even more complex force fields to deal with their structural complexity. This challenge leaves this research branch of cheminformatics very active.^82^ Friedrich *et al.*,^104^ compared seven free 3D conformer ensemble generators (RDkit DG algorithm, Experimental-Torsion basic knowledge distance Geometry algorithm (ETKDG), Confab, Frog2, Multiconf-DOCK, Balloon DG, and GA algorithms) to eight commercial counterparts (ConfGen, ConfGenX, cxcalc, iCon, MOE Low modeMD, MOE Stochastic, MOE Conformation Import, and OMEGA) using the Platinum Diverse Dataset. The Platinum Diverse Dataset is a high-quality benchmarking dataset of 2859 protein-bound ligand conformations extracted from the PDB.^104–106^ However, Chen *et al.*^97^ in 2018 characterized the chemical space of known and readily available NPs. They reported that of the over 250 000 structures of NPs available from public databases, only ∼2000 NPs were identified with at least one X-ray crystal structure of the compound in complex with a biomacromolecule available from the PDB.^97^ This is quite a very small data to be used as a representative for the entirety of NPs characterized so far.

Going back to the observations from Friedrich *et al.*,^104–106^ the best 3D conformer generators were the commercially available ones. Shortcomings of the free tools include critical errors in bond lengths, bond angles assignment and planarity or out-of-plane errors in the conformers generated. Although the commercially available algorithms were better, some abnormalities regarding geometries were also observed. All these lead to the conclusion that it is a research race to solve the challenge of an accurate algorithm capable of handling the diverse complexity of structures with ideas to include more structures of NPs.

## Starting structure (prodrugs)

Prodrugs represent a class of chemotherapeutics which remain inactive in the body until metabolized. The idea of prodrugs is to overcome pharmacokinetic and pharmacodynamic barriers (such as poor solubility, absorption, toxicity, side effects, and poor efficacy among other properties).^107^ This class of molecules (such as sulfasalazine, latanoprostene, psilocybin, aspirin, codeine, irinotecan, l-dopa, heroin, and several antiviral nucleosides) have enjoyed clinical successes over a long time for treating chronic and acute conditions.^108,109^ Prodrugs could occur naturally or they could be derived from semisynthetic processes or synthetic-designed intentionally during the rational drug design or unintentionally during the drug development.^109^

## Drug-likeness

The concept of drug-likeness focuses on the similarity of some physicochemical properties such as molecular mass, hydrophobicity, lipophilicity, polarity and hydrogen bond donors/acceptors.^110,111^ Analysis of these properties shows that approved drugs preferentially fall within a certain range of values and new compounds with physicochemical properties that fall within that range are likely to be considered as “drug-like”. Fascinatingly, *in silico*-based methods are being used to predict such properties from the molecular structure before the substance is even synthesized and tested. The rule of 5 (Ro5) and the quantitative estimate of drug-likeness (QED) discussed below represent a few of the approaches used to predict drug-likeness.

### Rule of five (Ro5)

Since its inception in a seminal work of Lipinski and co-workers in the late 90s,^111^ Ro5 has gained popularity in the medicinal chemistry community as guidelines to computationally estimate the “drug-likeness” of pharmacologically relevant molecules. This set of guidelines is summarized as follows: molecular weight (MW) < 500 Da, octanol/water partition coefficient (log *P*) < 5, H-bond donor (HBD) ≤ 5, and H-bond acceptor (HBA) ≤ 10. Generally, molecules with no more than one Ro5 violation are considered to be “drug-like” or orally available. Despite Lipinski's recommendation that the Ro5 be considered as a guideline, reality has it that it is used routinely to filter chemical libraries, especially during VS as a primary step. Since most NPs have complex structures with more than one Ro5 violation, they have significantly received less attention as potential lead compounds. They are underrepresented in ready-to-use VS libraries such as the ZINC library.^112^ This does not come as a surprise because the Ro5 did not consider NPs and substrates of biological transporters.

### Quantitative estimate of drug-likeness (QED)

Twelve years after the establishment of the Ro5, Bickerton *et al.*^110^ proposed an updated measure of drug-likeness based on the concept of desirability called the quantitative estimate of drug-likeness (QED). QED grades a molecule on a range from zero to one representing the favourability of the properties. Molecules with all the properties being unfavourable are graded zero while those with all the properties being favourable are graded one. To demonstrate the utility of this approach, QED was used to describe the desirability functions derived from a set of orally absorbed approved drugs as well as to solve the problem of molecular target druggability on a large set of published bioactive compounds. This approach compared favourably with rule-based metrics such as Lipinski's Ro5 (*e.g.*, at the levels of predictivity, intuitive and simplicity to implement). QED offers an even richer, more nuanced view of drug-likeness as it can identify cases in which a generally unfavourable property may be tolerated when the other parameters are close to ideal.

### Case study I: evaluating the drug-likeness of StreptomeDB 2.0

**Fig. 2:**
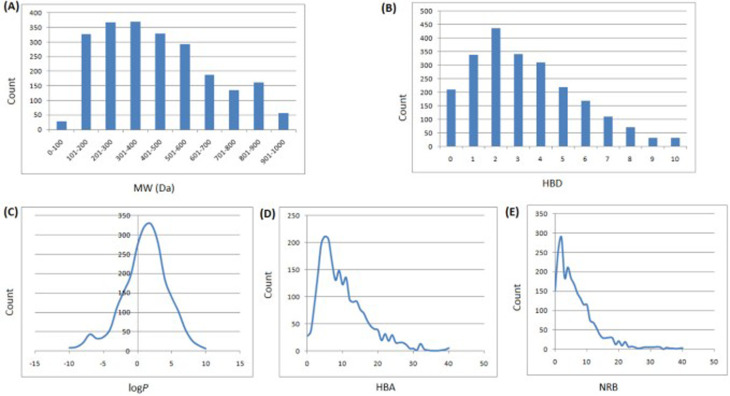
Ro5 evaluation of StreptomeDB 2.0. (A) MW, (B) HBD, (C) log *P*, (D) HBA, (E) NRB. The original figure was published under a Creative Commons License.

**Fig. 3:**
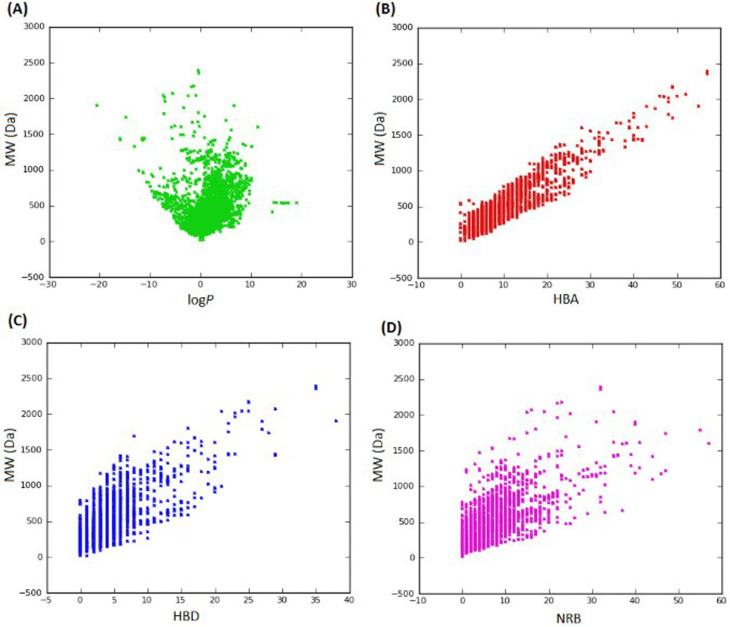
Pairwise comparison of Ro5 descriptors of StreptomeDB 2.0. (A) MW against log *P*, (B) MW against HBA, (C) MW against HBD and (D) MW against NRB. The original figure was published under a Creative Commons License.

In 2013, Ntie-Kang^113^ evaluated the drug likeness of about 2400 NPs of StreptomeDB 2.0; currently in its third version.^51,114^ StreptomeDB represents a database of NPs isolated from Gram-positive bacteria of the genus *Streptomyces*, constituting the largest source of clinical antibiotics. In addition to Ro5 descriptors, the number of rotatable bonds (NRB) was equally computed following the work of Veber *et al.*^115^ From this evaluation, it was observed that 52.5% of the compounds within StreptomeDB 2.0 had at least one Ro5 violation; meanwhile, 22.7% had more than two violations. Taken individually, MW was the descriptor exhibiting the most skewed distribution; approximately 42% of the molecules had MW > 500 Da (Fig. 2). Nevertheless, when pairwise compared in biplots, a trade-off in the interrelationships between these descriptors were observed by Ntie-Kang^113^ (Fig. 3).

It is worth mentioning that the vast majority of molecules with more than one Ro5 violation (beyond Ro5) in the StreptomeDB 2.0, are polyketides. This class of microbial NPs has previously yielded several important antibiotics such as streptomycin and erythromycin, anticancers such as bleomycin, and dactinomycin, and anthelmintics such as avermectins. But most newly isolated and characterized polyketides of this database remain pharmaceutically untapped.

### Case study II: drug-likeness evaluation of marine NPs

More recently, Pilkington performed a drug-likeness evaluation of a dataset of 179 marine NPs originating from three kingdoms (Animalia, Bacteria, and Fungi).^116^ Ro5 descriptors were computed as well as NRB, topological polar surface area (TPSA) and water solubility (log *S*). Based on defined thresholds, the authors grouped these NPs into three categories, namely, leadlike, druglike, and known drug spaces (Table 1).

**Table 1:** Classification of chemical spaces. Adapted from a paper published under a Creative Commons License^116^

Descriptor	Leadlike	Druglike	Known drug
MW	300	500	800
log *P*	3	5	6.5
HBD	3	5	7
HBA	3	10	15
TPSA	60	140	180
NRB	3	10	17

**Fig. 4:**
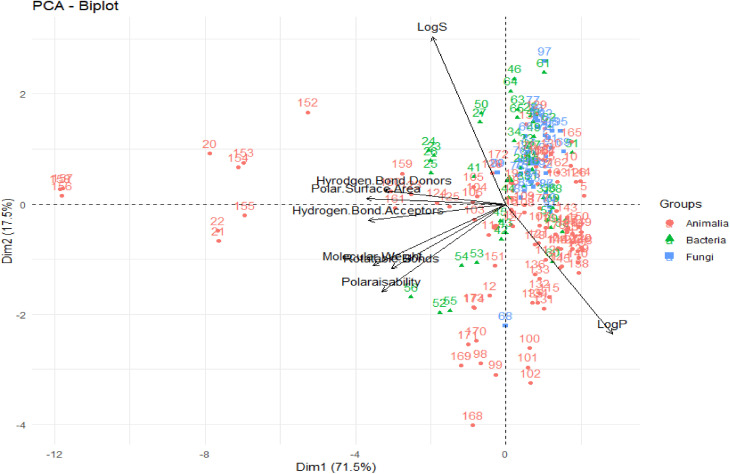
PCA of computed descriptors. The original figure was published under a Creative Commons License.

A PCA was performed to assess the interrelationships of computed descriptors. The two principal components of this analysis were the variability in the data (*x*-axis) and the dimensionality of variability (*y*-axis), with values of 71.5% and 17.5%, respectively (Fig. 4).

From this PCA analysis, they observed that except for log *S* and log *P*, all other descriptors greatly contributed to the variability. Moreover, log *P*, log *S*, and TPSA contributed the most to the second principal component. Overall, the chemical space distribution of these marine NPs in the “lead-like”, “drug-like” and known drug categories are 0.5%, 39.7%, and 64.8%, respectively. The contrast between the population of the known drug space of marine NPs, and that of the “drug-like” and “lead-like” spaces, rationalizes their underrepresentation in typical VS libraries.

## Hits optimization

The next step after the bioassay-guided isolation of bioactive NPs is the optimization of the hit molecule(s) using diverse strategies to improve their pharmacodynamic and pharmacokinetic properties, and in turn increase their biological activities.^103,117^ While Ro5 provides a quick overview of the oral availability of molecules of interest, a more comprehensive evaluation of their pharmacokinetics and toxicity profiles is generally required. *In silico* predictions of ADMET properties guide the prioritization of compounds for the more cost- and labour-intensive *in vitro* and/or *in vivo* preclinical pharmacokinetics evaluations. This in turn informs lead optimization and drug candidate selection. Some of the most popular software tools used for *in silico* ADMET predictions include pkCSM,^118^ ADMETlab,^119^ QikProp (Schrödinger, LLC, New York, NY),^120^ SwissADME^121^ and admetSAR.^122^ These tools rely upon quantitative structure–property relationship (QSPR) models built from experimentally available data of small-molecules.^123^ Although ADMET prediction tools have achieved great success for structurally diverse compounds, their reliability is put into question for predictions concerning NPs because these models are mainly trained and benchmarked on experimental data of structurally simple synthetic molecules. Hence, the selection of NPs for preclinical studies has been hampered by their undesirable predicted ADMET properties, despite displaying interesting biological activities in phenotypic screens. We briefly highlight a few of such cases.

### Case study I: ADMET evaluation of the Mexican NPs of the BIOFACQUI

**Fig. 5:**
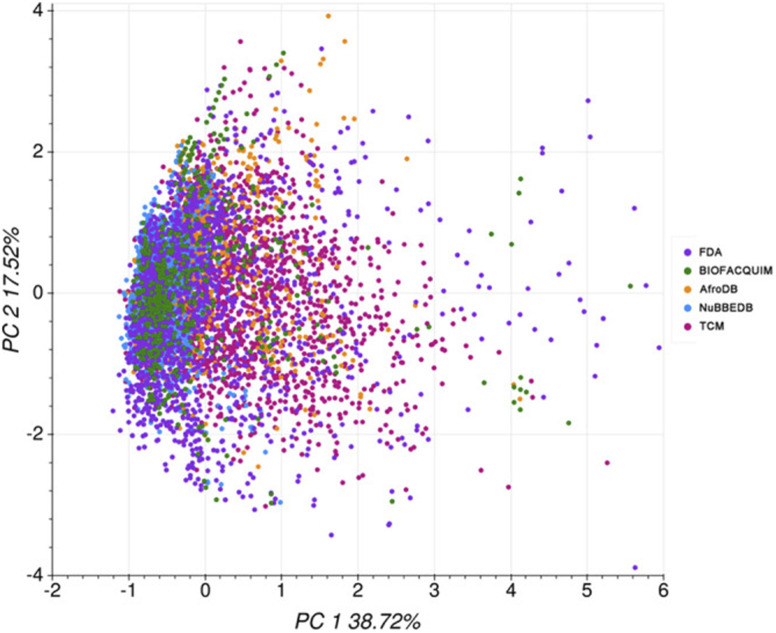
PCA on ADMET descriptors for selected compound datasets (variance = 56.2%). The original figure was published under a Creative Commons License.

In 2020, Medina-Franco and co-workers carried out a computational ADMET evaluation of the Mexican NPs of the BIOFACQUIM.^124,125^ A comparison was made with the profile of the African NPs of the AfroDB,^99^ the Brazilian NPs of NuBBEDB,^98^ the Chinese NPs of the TCM^126^ and the FDA-approved drugs dataset of the DrugBank.^127^ The authors used the pkCSM^118^ and the SwissADME^121^ webservers. From these predictions, it could be deduced that the absorption profile of BIOFACQUIM was similar to those of NuBBEDB, TCM and AfroDB; meanwhile, its distribution profile was similar to that of the FDA dataset based on the blood–brain barrier (BBB) permeability and the unbound fraction descriptors. Moreover, its metabolism profile was similar to those of AfroDB, TCM, NuBBEDB and the FDA dataset based on CYP1A2, CYP2D6, CYP3A4, and CYP2C19 inhibition, respectively. On the other hand, its excretion profile was more similar to that of TCM than that of the FDA dataset. Its toxicity profile was similar to that of the FDA dataset for the Human Ether-a-go-go-related Gene (hERG) II inhibition and Ames toxicity, and similar to that of TCM only for Ames toxicity. Its toxicity profile for hERG I inhibition is similar to those of AfroDB, NuBBEDB, and TCM. Lastly, its hepatotoxicity profile was similar to those of NuBBEDB and TCM. Altogether, based on a PCA on 16 ADMET descriptors, the chemical space coverage of each dataset was charted (Fig. 5).

### Case study II: ADMET profile of a dataset of protein–protein interface inhibitors

In 2017, Lagorce *et al.*,^128^ described the computed ADMET profile of a dataset of protein–protein interface inhibitors (iPPIs) collected from the IPPI-DB,^129^ TIMBAL,^130^ as well as that of a non-iPPIs dataset of orthosteric and allosteric inhibitors extracted from the ChEMBL database.^131^ The development of iPPIs is of growing interest because they address targets that are typically considered to be “undruggable”. The ADMET profiles of both constructed datasets were assessed with the pkCSM^118^ server in a similar fashion as described in the above example. Proteins that molecularly recognize other proteins have a typical binding sites, that is, large surface grooves that cannot be addressed by typical small-molecule ligands but rather by beyond-Ro5 molecules.^132^ The observations arrived at similar conclusions from their ADMET profiling. Numerous NP (macrocyclic) polyketides have been identified as very potent iPPIs.^133^ It is worth mentioning that Lagorce *et al.*^128^ excluded from the ADMET profiling all NP-(derived) molecules on the basis that this class of molecules are not equally included in training and/or benchmark datasets of ADMET prediction models such as that of pkCSM.^118^

## Sample availability

Physical samples of VS hits with interesting pharmacokinetic properties are needed for screening in wet laboratories against different assays of interest. These physical samples (pure compounds isolated from complex mixtures) form the basis of the standard methodology in NPs drug discovery. However, studies have shown that for a virtual collection of ∼250 000 NPs only about 10% of the collection is readily available for purchase.^46^ Also, the anti-biopiracy laws, including the Nagoya Protocol have been a major regulation for researchers involved in the bioprospecting of NPs from both the terrestrial and marine ecosystems.^134–136^ Adoption of the Convention on Biological Diversity (CBD), which came into force on December 29, 1993, aimed to promote sustainable use of biodiversity as well as conservation and benefit sharing of genetic resources. CBD restricts the collection and evaluation of plant, marine, and other samples by researchers. This amongst other factors, makes NP chemists face challenges in the isolation and purification of bioactive NPs from plants and marine organisms.^137^ In the majority of cases, the bioactive compounds are present in too low concentrations to be efficiently isolated, variation in the active constituents, loss of activity or failure in isolating the target bioactive compounds, degradation of heat labile compounds during the purification process; bioactivity was as a result of synergistic effects between multiple compounds.^103^ After the identification of some molecules as VS hits, the cost of collection of the samples (plants and access to the deep in the case of marine microorganisms) as well as synthesizing NPs with high structural complexity is expensive, tedious and time-consuming coupled with associated synthesis scale-up issues. A glimpse of a few of the mentioned challenges listed here for some *in silico*-based driven studies are discussed in the examples below.

### Case study I: repurposed use of taxol

**Fig. 6:**
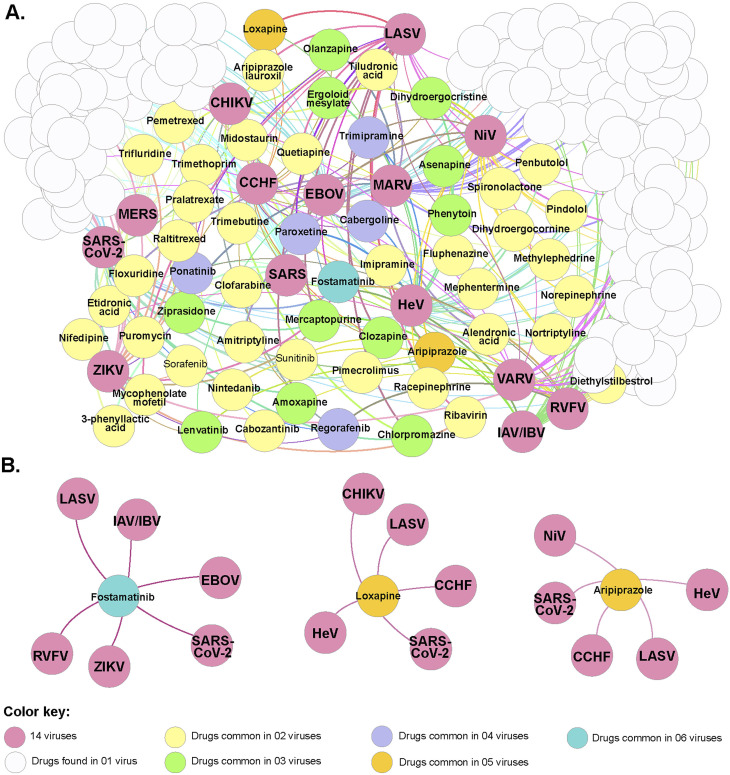
The network displayed common repurposed drugs between different viruses using the pipeline generated by Rajput *et al*.^146^ (A) Correlations between the repurposed drugs identified using the “drug-target-drug” approach and 14 viruses causing epidemics/pandemic. (B) Interaction diagram of identified repurposed drugs found in common for more than five viruses (figure reproduced with permission).

With the advent of the COVID-19 pandemic, several studies have been performed in other to suggest different specific treatment regimens as well as propose new antivirals with different therapeutic targets, that can help to reduce the level of morbidity and mortality.^138–145^ One such computational study to suggest drug candidates to treat a viral infection was reported by Rajput *et al.*^146^ In the study, several drug molecules were suggested using drug-target network analysis for repurposing drug molecules as potential antivirals to combat epidemics and pandemics (Fig. 6). One such molecule is paclitaxel (also known as Taxol Fig. 1) an effective agent against the influenza virus. Taxol is an approved molecule for the treatment of Kaposi's sarcoma and lung cancer (by the US FDA) and other studies have equally suggested that it could treat skin disorders, renal and hepatic fibrosis, inflammation, axon regeneration, limb salvage, influenza and coronary artery restenosis at low concentrations. However, the suggestion of Taxol as a potential antiviral agent comes with challenges as it is difficult to obtain meaningful quantities of this bioactive NP for biological screening assay as well as for commercial supply globally.^147^

### Case study II: evaluating the histone deacetylase (HDAC) inhibitory activities of NPs from the African NP database collection

**Fig. 7:**
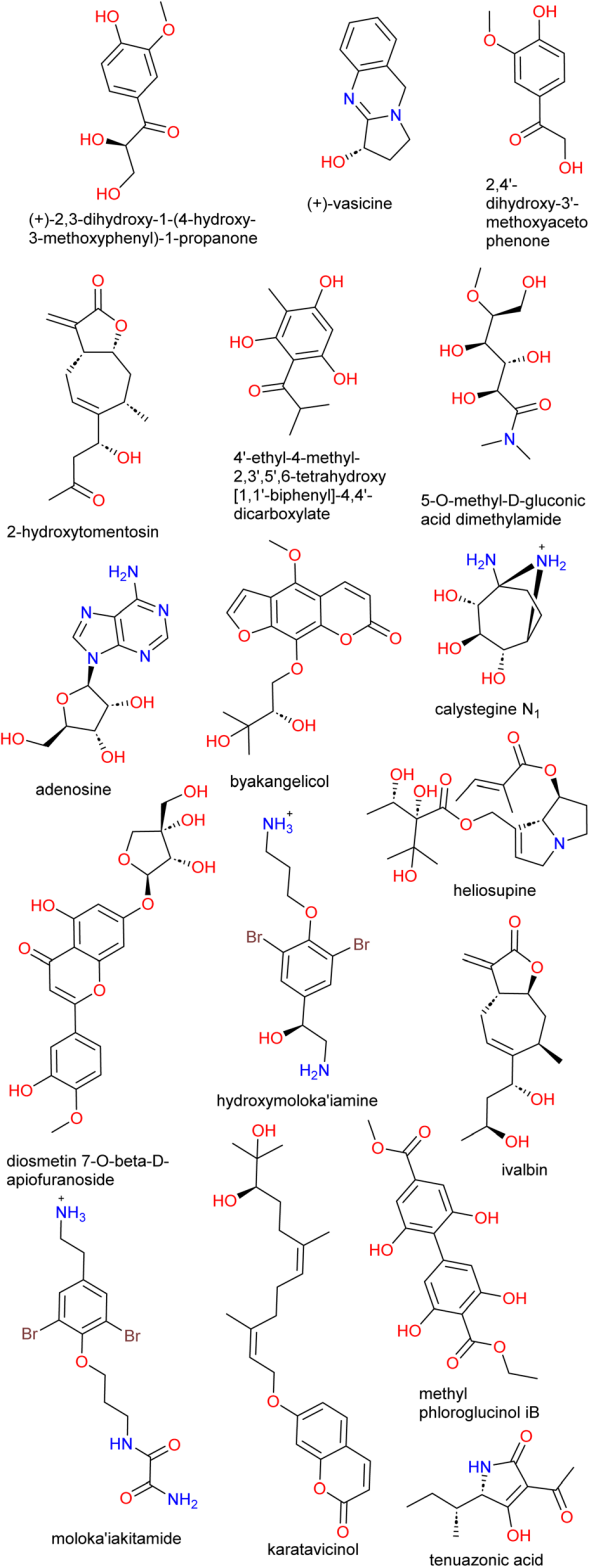
Virtual hits from ANPDB as HDAC inhibitors.

Another example of a work that highlights the challenges in getting and testing NP samples after *in silico* studies is reported by Simoben *et al.*^148^ In this study, several computational methods coupled to biological testing were applied to suggest novel inhibitors of HDAC isoforms.^148–150^ HDACs represent an interesting class of zinc-dependent enzymes with very important physiological roles that are linked to several diseases such as cancer, cardiac and neurodegenerative diseases, inflammation, metabolic and immune disorders, and viral and parasitic infections.^151–155^ Interestingly, about two hundred molecules inspired by nature have been investigated as HDAC inhibitors.^156–166^ Analysis of a database of NPs from African source species showed that they occupy chemical spaces that were not previously reported in published NP databases as well as compounds that are similar to HDAC inhibitors. After the VS of the said database, a set of molecules was proposed as hits (Fig. 7). However, of the seventeen molecules suggested, only three were experimentally tested due to the difficulty in obtaining physical samples or the extravagant cost of some of the hits. Thus, the proposed hits may act as a starting point in a structure-based design and/or in chemical optimization efforts to improve the suggested novel HDAC inhibitors. Also, the study supports the idea that *in silico* approaches can readily identify novel HDAC modulators.

### Case study III: *in silico* identification of bichalcones as sirtuin inhibitors

**Fig. 8:**
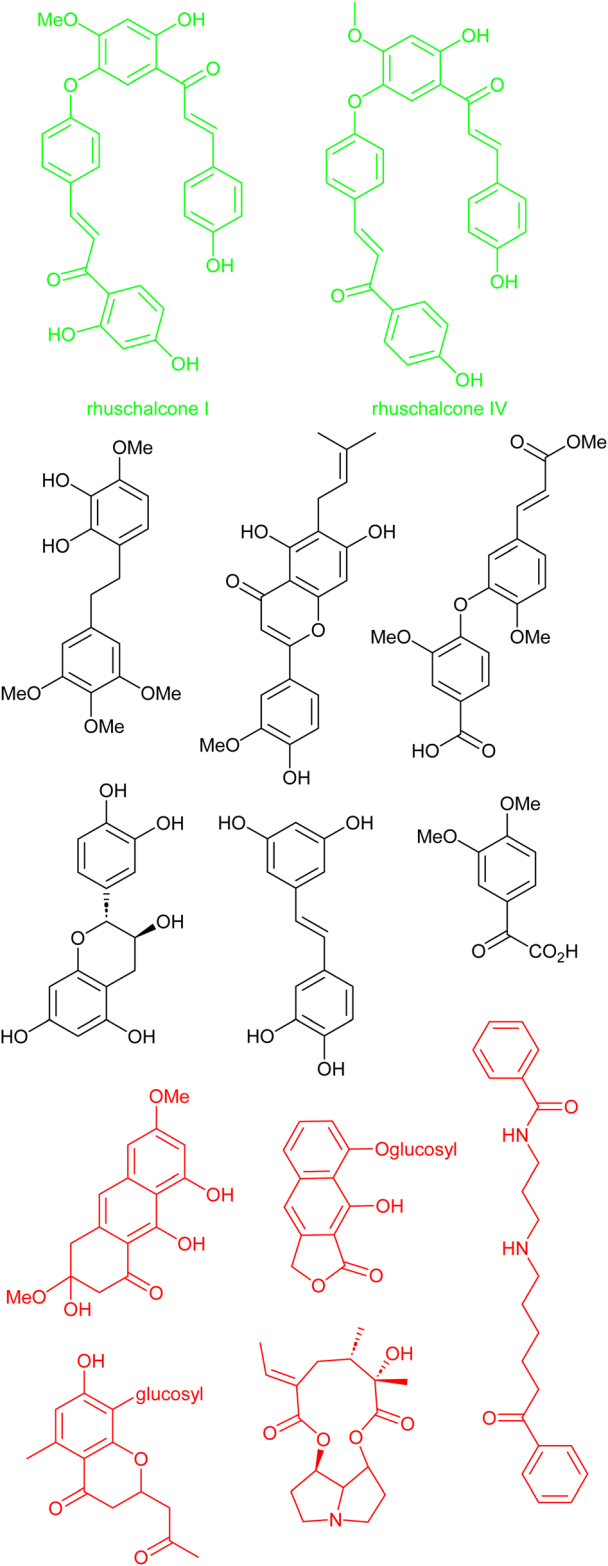
13 out of the 22 suggested virtual hits: black (>1 mg), red (tested negative) and green (active).

Another interesting study by Karaman *et al.* shows that there is a challenge in obtaining physical samples of NPs involved in the *in silico*-based approach to identify bichalcones as sirtuin inhibitors.^167^ Sirtuins represent a subset (Class III) of histone deacetylases and are characterized as nicotinamide adenine dinucleotide (NAD+)-dependent.^168^ Sirtuins have been linked to several diseases, including cancer, HIV, and neurodegeneration.^168–171^ With the attention NPs have received in the quest to search for sirtuin inhibitors. Karaman *et al.* performed VS using the pan-African Natural Products Library (p-ANAPL)^172^ (a collection of physical samples of NPs from African source species) to suggest hit molecules as sirtuin inhibitors. From the virtual study, 22 hits were proposed (Fig. 8). However, only five compounds (about 25% of the suggested hits) had sufficient quantities to further investigate their *in vitro* activity as sirtuins inhibitors. Of the five molecules biologically evaluated, two of them (the bichalcones rhuschalcone IV and an analogue of rhuschalcone I (Fig. 8); isolated from the medicinal plant *Rhus pyroides*) showed *in vitro* activity. The results therefore showed that these molecules could represent a class of compounds that can be optimized to improve the biological activities as sirtuins inhibitors. Additionally, the authors also provided suggestions based on *in silico*-based studies, on how the biological activities could be improved.

## Intellectual property

Identification of virtual hits with interesting pharmacokinetic properties needs to be confirmed through different biological screening assays in the wet laboratories using the physical samples. Sadly, accessing sufficient quantities of the identified virtual hits for characterisation and biological assaying may also be challenging. The Nagoya Protocol has disadvantaged NP researchers.^134–136^ This hurdle even restricted researchers from the collection and evaluation of plant and marine samples after the adoption of the Convention on Biological Diversity (CBD), which came into force on December 29, 1993, to promote sustainable use of biodiversity as well as conservation and benefit sharing of genetic resources.^136,173^ Further complexities are observed at the levels of (i) filling in for intellectual property (IP)^174^ rights when it comes to (unmodified) NPs exhibiting very interesting bioactivities and (ii) the benefit sharing due to regulations defined by the countries of origin where the biological material was collected (especially when it concerns marine genetic samples).^134–136^

## Conclusions

NPs have played an important role in improving the living conditions of humans as well as in the treatment of ailments. Despite the many examples of advantages and success stories recorded for NP drug discovery, several challenges encountered have led pharmaceutical companies to reduce programmes in this sector. This review is intended to highlight the challenges that are encountered when applying *in silico*-based methods in the search for new drug candidates from a NP perspective. Some of the challenges discussed herein include but are not limited to the computational power and skills, to access and explore the numerous and large data collection of NP databases and, the availability of physical samples for compounds identified as hits in reasonable quantity. Having an idea of the discussed challenges would prepare the minds of interested scientists in this area of research as well as give them an orientation.

## Author contributions

C. V. S., W. S., J. L. M.-F., and S. G. conceived the idea. All authors were involved in the writing and compilation of the manuscript.

## Conflicts of interest

There are no conflicts to declare.

## Supplementary Material
